# From canines to humans: Clinical importance of *Staphylococcus pseudintermedius*

**DOI:** 10.1371/journal.ppat.1009961

**Published:** 2021-12-02

**Authors:** Karen C. Carroll, Carey-Ann D. Burnham, Lars F. Westblade

**Affiliations:** 1 Division of Medical Microbiology, Johns Hopkins Hospital, Baltimore, Maryland, United States of America; 2 Department of Pathology and Immunology, Washington University in St. Louis School of Medicine, St. Louis, Missouri, United States of America; 3 Department of Pathology and Laboratory Medicine, Weill Cornell Medicine, New York, New York, United States of America; Nanyang Technological University, SINGAPORE

## Introduction

Historical observations led to the development of 2 important diagnostic assays: the classic tube coagulase test (in which fibrinogen is converted to fibrin by staphylocoagulase or von Willebrand binding protein, *i.e.*, “free coagulase”) and the slide coagulase assay (whereby fibrinogen is bound by clumping factor, *i.e.*, “bound coagulase”) [1,2]. These assays (or derivatives of) are used to differentiate staphylococcal isolates that elaborate coagulase: coagulase-positive *Staphylococcus* species (CoPS), and those that do not: coagulase-negative *Staphylococcus* species (CoNS). This is an important distinction because, in general, CoPS are considered to be more pathogenic than CoNS.

**Fig 1:**
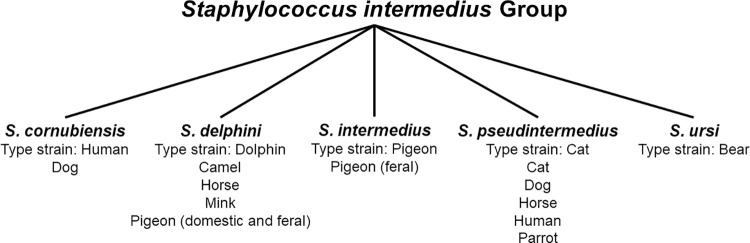
Members of the *S. intermedius* group and host associations. Host associations based upon data presented in [5,6,67,70]. The animal sources of the type strains (*Staphylococcus cornubiensis* NW1^T^, *Staphylococcus delphini* DSM 20771^T^, *Staphylococcus intermedius* DSM 20373^T^, *Staphylococcus pseudintermedius* DSM 21284^T^, and *Staphylococcus ursi* MI 10-1553^T^) are indicated, and host associations of the different species are listed underneath. The human isolates likely reflect transmission from animal sources. In the case of *S*. *cornubiensis*, the type strain was recovered from a human [67]; however, a related isolate (2008-01-1056-2) was recovered from a dog [71], suggestive of an animal link. All members, with the notable exception of *S*. *ursi*, are coagulase positive.

*Staphylococcus aureus* is the archetype CoPS and an important human pathogen [3]; however, several CoPS, most associated with animals, have been described [1,2] (Table 1). One CoPS of emerging clinical importance is *Staphylococcus pseudintermedius*, a member of the *Staphylococcus intermedius* group (SIG) (Table 1 and Fig 1). *S*. *pseudintermedius* is a significant pathogen in canines, an agent of infections in humans, and is the focus of this “Pearl”.

**Table 1:** CoPS.

*Staphylococcus* species/subspecies	Free (tube) coagulase activity^a^
*Staphylococcus agnetis*	+
*Staphylococcus argenteus* ^b^	+
*Staphylococcus aureus* subspecies *anaerobius*^b^	+
*S*. *aureus* subspecies *aureus*^b^	+
*Staphylococcus chromogenes*	+
*Staphylococcus coagulans* ^c^	+
*Staphylococcus condimenti*	+
*Staphylococcus cornubiensis* ^d^	+
*Staphylococcus delphini* ^d^	+
*Staphylococcus hyicus*	+
*Staphylococcus intermedius* ^d^	+
*Staphylococcus lutrae*	+
*Staphylococcus pseudintermedius* ^d^	+
*Staphylococcus schweitzeri* ^b^	+

^a^ Coagulase reactivity taken from [1,2,67]. Free (tube) coagulase activity reflects staphylocoagulase activity (which is exclusively associated with *S*. *argenteus*, *S*. *aureus* subspecies *aureus*, and *S*. *schweitzeri*) and von Willebrand binding protein activity in either avian, canine, equine, human, porcine, or rabbit plasma.

^b^ Member of the *S*. *aureus* complex [68].

^c^ Formerly *Staphylococcus schleiferi* subspecies *coagulans* [69].

^d^ Member of the *S. intermedius* group. One member, *Staphylococcus ursi*, recovered from black bears, is coagulase negative and thus not included [70].

+, positive; CoPS, coagulase-positive *Staphylococcus* species.

## What is the epidemiology of *S*. *pseudintermedius*?

The taxonomy and host associations of SIG members have been redefined by molecular typing methods (Fig 1). In 2 seminal studies, all isolates recovered from dogs, cats, and humans were identified as *S*. *pseudintermedius*, and thus *S*. *pseudintermedius* (not *S*. *intermedius*) was revealed as the common agent of canine pyoderma [4–6]. It is a component of the normal skin microbiota of dogs, and one review cites, based upon the literature, canine carriage rates per body site of: nose, arithmetic mean 31% (range, 16% to 64%); mouth, arithmetic mean 57% (range, 42% to 74%); perineum–rectum, arithmetic mean 52% (range, 28% to 72%); and groin, arithmetic mean 23% (range, 16% to 38%) [7]. Carriage rates in noncanine species, such as cats and horses, appear to be much lower than in dogs, with only 6.8% of cats colonized compared to 46.2% of dogs [8,9].

Humans are not the natural host but can be transiently colonized with *S*. *pseudintermedius*, including methicillin-resistant *S*. *pseudintermedius* (MRSP); however, the prevalence of human colonization is unknown as this organism can be misidentified as *S*. *aureus* [10,11]. In one study, enriched with individuals involved in the veterinary profession (42.5% of study participants), the prevalence (nasal colonization) of *S*. *pseudintermedius* in humans living in a household with a dog or cat was 4.1% (by contrast, the prevalence of *S*. *aureus* in humans was 27.7%), with lack of handwashing after handling household pets significantly associated with colonization [8]. In a subsequent study, 3.9% of small animal dermatologists surveyed were found to be colonized in the nares with MRSP [12]. Finally, Guardabassi and colleagues demonstrated that owners of dogs with pyoderma were more likely to be culture positive for *S*. *pseudintermedius* compared to persons without daily contact with dogs, but colonization was not documented at the time of a second sampling 2 months later, suggesting that long-term colonization is uncommon in humans (although dogs were treated with antimicrobials after the first sampling, and most no longer had purulent lesions at the second sampling) [13].

## What are the clinical manifestations of *S*. *pseudintermedius* in animals and humans?

Infections predominantly involve the skin and skin structures (SST) of canines. SST infections (SSTIs) range from superficial bacterial folliculitis (SBF), characterized by multifocal areas of alopecia, follicular papules or pustules, serous crusts on the trunk and axillary areas, and epidermal collarettes to deeper infections such as furunculosis and cellulitis [7,14,15]. *S*. *pseudintermedius* is also a cause of otitis and urinary tract infections in dogs [16,17]. In a large case series from Italy [16], *S*. *pseudintermedius* constituted 76% of all *Staphylococcus* species recovered from dog and cat cultures, and 31.6% of those were MRSP. The most common presentations for MRSP isolates were otitis (36.8%), pyoderma (21%), cutaneous fistulae (15.8%), conjunctivitis (10.5%), postsurgical infections (10.5%), and prostatitis (5.3%). Almost all of the infections were seen in dogs, and the MRSP isolates belonged to several clones, most notably sequence type (ST) 71, with variable multidrug resistance (MDR) determinants.

The most common manifestations of infections in humans are SSTIs including dog bite wounds [10,11,18,19]. Somayaji and colleagues described one of the largest series of human *S*. *pseudintermedius* infections among 24 patients (27 isolates) [19]. SSTIs that occurred in 18 patients and 2 invasive infections, including a bloodstream infection and a prosthetic joint infection, were described. After chart review, 4 patients were determined to be colonized and not infected. Diabetes, peripheral vascular disease, and cardiovascular disease were the most significant comorbidities among this cohort, and 95.4% of patients had contact with dogs. Most of the infections were polymicrobial (91.7%), and the infections were primarily mild to moderate in severity, allowing the patients to be managed in the outpatient setting. A total of 3 patients were infected with MRSP isolates, and all were MDR. These MDR isolates belonged to MRSP lineages ST71 and ST181. Serious wound infections in patients, including diabetic patients, caused by the specific *S*. *pseudintermedius* epidemic clone ST71-J-t02-II-III, were described by Starlander and colleagues [18]. Interestingly, the authors suggested indirect or direct patient-to-patient acquisition of the epidemic clone between patients was very likely. No animal source could be identified. Yarbrough and colleagues also documented several instances where *S*. *pseudintermedius* was recovered from human clinical specimens without documented animal exposure, although it is unclear if this reflects human-to-human transmission or an incomplete medical history [11].

Human respiratory infections (sinusitis, otitis, and pneumonia) are well described in the literature [11,19–22]. Kuan and colleagues described symptoms and findings in 4 patients with serious rhinosinusitis, and they reviewed the literature on this entity [21]. Symptoms of chronic rhinosinusitis occurred in all patients. A total of 3 of 4 patients had immunocompromising conditions. Sinus cultures revealed mixed pathogens, and prolonged antimicrobial therapy and surgical debridement were required for treatment. In a series of 33 patients with chronic rhinosinusitis, there was no difference in immunocompromising factors between patients with cultures positive with *S*. *pseudintermedius* and those that had other organisms [22], although dog ownership was more common in the *S*. *pseudintermedius* group. In addition, 27% of the isolates were MRSP and the authors emphasized the importance of identifying the etiology of rhinosinusitis and determining antimicrobial susceptibility to guide therapy even in immunocompetent patients.

The invasive potential of *S*. *pseudintermedius* in humans has been demonstrated in several case reports/series of bacteremia, endocarditis, and device-associated infections [19,23,24]. In a striking case report, Darlow and colleagues presented a case of invasive spinal infection in a patient with hardware who had chronic open skin lesions that served as the portal of entry for the organism associated with the patient’s dog. Ensuing bacteremia likely infected the spinal fixation devices in place from a prior surgery [24].

## What is the basis for *S*. *pseudintermedius* pathogenicity?

*S*. *pseudintermedius* virulence factors include cytotoxins, exfoliative toxins, superantigens, and cell wall–associated (CWA) proteins and are important in initiating and spreading infections such as SSTIs and evading the immune system, and many are orthologous to those elaborated by *S*. *aureus* [7,14,25,26]. Additionally, *S*. *pseudintermedius* has the ability to form biofilms [27]. Enzymes that play an important role include proteases and thermonucleases, and the promotion of plasma coagulation (*i.e.*, coagulase activity) by von Willebrand binding protein is a key pathogenic trait [2,7,14,26]. *In silico* analysis of the *S*. *pseudintermedius* ED99 genome led to the prediction of 18 putative CWA proteins termed “*S**taphylococcus* *p**seudintermedius* surface proteins” (Sps) [25]. These proteins endow *S*. *pseudintermedius* with adherence properties and 2, SpsD and SpsL, facilitate adherence to proteins of the host extracellular matrix: fibronectin, fibrinogen, and cytokeratin 10 [28]. In a study that examined bone and joint infection pathophysiological mechanisms among non-*S*. *aureus* species, only *S*. *pseudintermedius* was able to adhere to human fibronectin in a bacterial adhesion assay, and adhesion rates were similar to those observed for *S*. *aureus* [29]. Protein A, a CWA surface protein, is a major *S*. *aureus* virulence factor that facilitates immune evasion by binding immunoglobulins, inhibiting opsonization and phagocytosis, and acts as a superantigen [26]. SpsP and SpsQ are *S*. *pseudintermedius* protein A orthologues and presumably serve an important role in pathogenesis given isolates recovered from infected dogs synthesize protein A more frequently compared to those recovered from healthy dogs [28,30,31].

Similar to *S*. *aureus* Panton-Valentine leukocidin, *S*. *pseudintermedius* produces a bicomponent leukotoxin (Luk-I) that is leukotoxic to polymorphonuclear white blood cells [32,33]. α-hemolysin and β-hemolysin are produced by *S*. *pseudintermedius* and can cause hemolysis of rabbit erythrocytes and hot–cold hemolysis of sheep erythrocytes [27,34,35]. The exfoliative toxin, *Staphylococcus intermedius* exfoliative toxin (SIET), plays an important role in canine pyoderma and dogs injected with SIET developed erythema, exfoliation, and crusting, which are characteristics of canine pyoderma [36]. Additional exfoliative toxins include ExpA and ExpB [37,38]. SEC_CANINE_ an enterotoxin, with variable prevalence in the *S*. *pseudintermedius* population and distinct from other staphylococcal enterotoxins, has the ability to induce vomiting and T-cell proliferation [39,40] Finally, similar to *S*. *aureus*, *S*. *pseudintermedius* has an accessory gene regulator (*agr*) quorum-sensing system that regulates expression of virulence factors in a population density–based manner [41].

Interestingly, of a variety of virulence factors assayed (*e.g.*, β-hemolysin, clumping factor, coagulase, DNase, protein A, and lipase), only protein A production was significantly different between *S*. *pseudintermedius* isolates recovered from healthy and infected dogs and was expressed in more isolates recovered from infected animals (1.4% versus 14.2%) [30]. Therefore, host factors likely play an important role in disease and disease severity. Predisposing factors to SSTIs in dogs include hypersensitivities, ectoparasites, endocrinopathies, and cornification abnormalities [15]. Other factors that may contribute to the development of infection in colonized dogs include medical/surgical procedures or immunosuppressive disorders [7].

## How is *S*. *pseudintermedius* treated?

SBF, the most common form of canine pyoderma and predominantly a result of *S*. *pseudintermedius* infection, is the primary reason for antimicrobial use in small animal practice [42–44]. Guidelines for managing canine SBF have been developed [45]. Conversely, no guidelines exist for managing human *S*. *pseudintermedius* infections. However, given its ability to elaborate toxins and virulence factors orthologous to those produced by *S*. *aureus*, human infections are often managed based upon culture-directed therapy.

A significant challenge to treating *S*. *pseudintermedius* is the emergence of methicillin (β-lactam) resistance, which results from acquisition of penicillin-binding protein 2a (PBP2a; encoded by *mecA*) [46]. Recent (within 1 month) administration of β-lactams or fluoroquinolones is associated with development of MRSP infection in canines [47]. Since the initial phenotypic description of MRSP in the 1980s [48], the frequency of MRSP has increased at an alarming rate. In one academic veterinary medical center in the United States (US), MRSP rates increased from <5% in 2001 to approximately 30% in 2008 [49] and, anecdotally, is reported to have reached 40% in some regions of the US [50]. MRSP prevalence in the human population remains largely unknown possibly because of potential misidentification as *S*. *aureus*, and breakpoints for detecting *mecA*-mediated resistance in *S*. *aureus* are inaccurate for *S*. *pseudintermedius* and can result in false susceptibility [10,11,49,51].

MRSP dissemination is primarily driven by organisms belonging to ST68 and ST71 [50,52,53], and is concerning as these organisms are often MDR and typically only susceptible to agents restricted or rarely considered for use in veterinary medicine (*e.g.*, daptomycin, linezolid, and vancomycin) [50,54,55]. Humphries and colleagues determined the antimicrobial susceptibility of 115 SIG isolates recovered from animals (56 canine isolates) and humans (*n* = 45) to 15 antimicrobials [55]. A total of 37 isolates harbored *mecA* and compared to *mecA*-negative isolates had decreased susceptibility to ciprofloxacin, clindamycin, doxycycline, erythromycin, and trimethoprim-sulfamethoxazole. Strikingly, human methicillin–resistant *S*. *aureus* isolates are often susceptible to doxycycline and trimethoprim-sulfamethoxazole [56,57]. This difference may facilitate identification in diagnostic laboratories where accurate identification methods are not available.

Given the dwindling armamentarium for treating MRSP, alternative treatment modalities that can be used safely in the canine population without compromising the efficacy of agents reserved for human infections are desperately needed. As infections predominantly involve the SST, potential therapies include honey-based gels [58] and bacteriophages [59,60]. Additional areas of investigation involve repurposing antimicrobials (*e.g.*, fosmidomycin) [61], and appropriation of nonantimicrobial agents has also garnered interest. For example, carprofen, a nonsteroidal anti-inflammatory drug, appears to restore doxycycline susceptibility in MRSP isolates carrying *tetK*, while chemotherapeutic agents commonly used in veterinary oncology (*e.g.*, bleomycin) can inhibit *S*. *pseudintermedius* growth [62,63]. Finally, candidate components for a vaccine have been proposed and include *S*. *pseudintermedius* toxins and virulence factors: Luk-I, protein A, and SpEX [64–66].

## Summary

*S*. *pseudintermedius* colonizes and infects animals and is a zoonotic pathogen. Infections in canines are primarily SSTIs, and, in many cases, dogs have predisposing factors leading to infection. Human infections are also predominantly SSTIs and often, but not always, with documented canine involvement. The invasive potential of *S*. *pseudintermedius* and its complement of virulence factors—many of which mirror those associated with *S*. *aureus*—demonstrate this organism has the potential to cause serious disease. β-lactam resistance has emerged as a serious problem for canine health and is typically associated with MDR. Therefore, given the close relationship between canines and humans, *S*. *pseudintermedius* is an important candidate for One Health initiatives.
